# Lasting Differential Effects on Plasticity Induced by Prenatal Stress in Dorsal and Ventral Hippocampus

**DOI:** 10.1155/2016/2540462

**Published:** 2016-01-10

**Authors:** Gayane Grigoryan, Menahem Segal

**Affiliations:** ^1^Division of Cellular Neurobiology, Zoological Institute, Technische Universität Braunschweig, Spielmannstrasse 7, 38106 Braunschweig, Germany; ^2^Department of Neurobiology, Weizmann Institute of Science, 76100 Rehovot, Israel

## Abstract

Early life adversaries have a profound impact on the developing brain structure and functions that persist long after the original traumatic experience has vanished. One of the extensively studied brain structures in relation to early life stress has been the hippocampus because of its unique association with cognitive processes of the brain. While the entire hippocampus shares the same intrinsic organization, it assumes different functions in its dorsal and ventral sectors (DH and VH, resp.), based on different connectivity with other brain structures. In the present review, we summarize the differences between DH and VH and discuss functional and structural effects of prenatal stress in the two sectors, with the realization that much is yet to be explored in understanding the opposite reactivity of the DH and VH to stressful stimulation.

## 1. Introduction

Evidence has accumulated in recent years to indicate that early life adversaries have a profound impact on the developing brain structure and functions, long after the original traumatic experience has vanished. One of the extensively studied structures in the brain in relation to early life stress has been the hippocampus. It is a unique structure in that it forms rather late in embryonic life and continues morphogenesis early in postnatal life [1–3]. The hippocampus is a focus of attention because of its unique association with cognitive processes of the brain. However, most studies describe effects of behavioral manipulations on structure/function of the dorsal hippocampus, but there are strong indications that while the entire hippocampus shares the same intrinsic organization, it assumes different functions in its dorsal and ventral sectors (DH and VH, resp.). The two sectors have different connectivity with other brain structures, and they differ in distribution of receptors, which leads to differences in function, different sensitivity, and very often opposite reactions to the same stimulus. In the following review, we will summarize the differences between DH and VH and we will carry on by describing some differential effects of stress in the two sectors, with the realization that much is yet to be explored towards understanding the opposite and long-lasting reactivity of the two sectors to stressful stimulation.

## 2. Ventral Hippocampus: Is It Different from the Dorsal Hippocampus?

**Figure 1:**
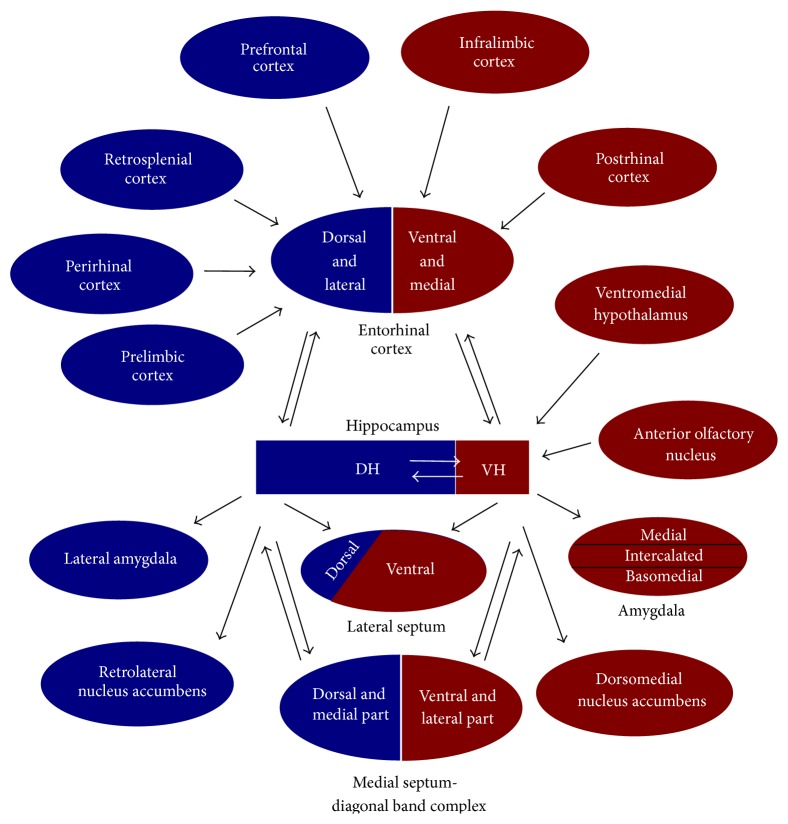
A schematic diagram of the major connections of the dorsal (DH) and ventral (VH) sectors of hippocampus.

The hippocampus has a curved shape that is conserved across all mammals and is distributed from dorsal (= septal, also called posterior in humans) to ventral (= temporal, anterior in humans) poles. The dorsal and ventral sectors of hippocampus (DH and VH, correspondingly) have different connectivity with cortical and subcortical structures, with the intermediate hippocampus sharing some properties with the DH. VH has more dense connectivity with the amygdala and hypothalamic endocrine and autonomic nuclei than DH [4, 5]. The VH projects preferentially to the medial, intercalated, and basomedial nuclei of amygdala and the amygdala-hippocampal transition area, while the DH distributes its efferents in more lateral regions of the amygdala [6]. The projections from cingulate areas (infralimbic and prelimbic cortices) involved in emotional regulation primarily reach the VH via input to the ventromedial parts of the entorhinal cortex (EC), while the anterior cingulate and retrosplenial cortices involved in spatial processing primarily project to the DH via targeting dorsal and lateral parts of the EC. Projections from the hippocampus to the EC originate in CA1 region and the subiculum and show a topographical organization similar to that of the EC-hippocampus inputs [7, 8]. The projection of the major hippocampal output to the lateral and medial septum (LS and MS, correspondingly) also shows dorsoventral differentiation. Thus, the DH projects to the small dorsal part of LS and dorsal and medial parts of MS, while VH innervates the larger ventral part of LS and lateral and ventral parts of MS [9, 10] (Figure 1).

The special character of the hippocampal connectivity forms a difference in the neurotransmitter composition along the axis of the hippocampus. Thus, cholinergic [11] and dopaminergic [12] innervation is denser in VH. Likewise, the concentrations of norepinephrine [13] and serotonin [14] as well as the density of synaptic terminals containing these transmitters are higher in the VH [15]. This differential distribution of several neuromodulators indicates that the VH is more amenable to neuromodulation than the DH.

Differences in the connectivity of DH and VH determine their functional distinction. Cross-species data show differences in connectivity with cortical and subcortical structures and functional differentiation along the longitudinal axis of hippocampus. This suggests that functional differences along the long axis may exhibit a gradient-like organization [16, 17] but that there are other connections that are restricted to the DH or VH. It has been suggested that the DH plays a crucial role in spatial learning and memory processes and VH is involved in anxiety, fear, defensive behavior, and stress related responses [18–24]. Studies with an animal model of hippocampal damage showed that lesions in DH impair spatial learning on tasks such as the Morris water maze or elevated T maze while lesions in VH disrupt emotional responses without impairments of spatial learning [25–29]. Massive activation of the DH during tasks that require spatial working memory was demonstrated by cFos staining as well [30]. Interestingly, NR1 N-methyl-D-aspartate (NMDA) receptor subunit deletion from the granule cells of the dentate gyrus (DG) not only impairs short-term spatial memory but also reduces anxiety [18]. The importance of VH in anxiety-related behavior such as anorexia nervosa was also shown [31]. Neonatal excitotoxic lesions of VH in rats result in postpubertal hyperresponsiveness to stress and cognitive abnormalities characteristic to those described in schizophrenia (for review, see [32, 33]). The anterior hippocampus in humans also shows anatomical [34] and functional [35] abnormalities in patients that had suffered from schizophrenia.

Single neurons in the DH and VH vary in their electrophysiological properties. While neurons recorded from CA1 area of DH and VH have similar spike discharge characteristics and could be classified into “complex spike” and “theta” cells, less than one-fourth of cell population in VH have “place” properties, and these have low spatial resolution, while in DH they represent at least half of the cell population with much smaller and better tuned place field size [36].

With respect to evoked field potentials and their plastic properties, in particular, their ability to undergo short- or long-term potentiation or depression (STP, LTP, and LTD, resp., major cellular mechanisms that underlie learning and memory processes [37]), DH and VH exhibit different properties as well. Examination of different forms of synaptic plasticity uncovered an impaired ability of VH to produce STP and LTP [38–41] and weaker synaptic inhibition with lower levels of gamma-aminobutyric acid (GABA) receptor A subunits [42, 43], which makes the VH more vulnerable to epileptic activity [43, 44]. The low ability of VH to express LTP might be due to its biochemical characteristics. Thus, DH and VH are different in the distribution of different subunits of NMDA receptors: the density of both NR2A and NR2B subunits of NMDA receptors is higher in DH than in VH [45, 46]. Moreover, the VH has lower levels of mRNA expression for GluRA, GluRB, and GluRC subunits of *α*-amino-3-hydroxy-5-methyl-4-isoxazolepropionic acid (AMPA) receptors compared with DH [46].

There is evidence for selective activation of different corticosteroid receptors in DH and VH in response to acute stress exposure. The activation of mineralocorticoid receptors leads to facilitation of LTP by enhancement of voltage-gated Ca^2+^ channels in VH, whereas the suppressed LTP in DH is mediated by activation of a glucocorticoid receptor [38]. Moreover, the VH differs from the DH in its sensitivity to agents that release Ca^2+^ from its internal stores (caffeine/ryanodine). Thus, VH exhibits higher sensitivity to ryanodine than DH which results in a strong response to subthreshold stimulation and is based on a higher level of Ca^2+^-store-related ryanodine receptors in VH [47, 48].

## 3. Changes of Synaptic Plasticity in Dorsal versus Ventral Hippocampus

Early life adversaries have an impact on health status and quality of life of individual and society at large; stress during pregnancy also has a profound role in the determination of the destiny of fetus. There are a number of protocols utilized in different laboratories to model prenatal stress (PS) in animals. Simulating PS in laboratory animals and especially interpreting and comparing the results from different groups demand a special care due to many factors related to the nature of the stressor(s) (type, duration, and “severity”), the “time window” during the pregnancy during which stress is experienced, genotype (wild type or genetically manipulated), and species (i.e., mouse, rat, guinea pig, or monkey) of pregnant dams as well as the age and sex of assessed offspring. In the majority of publications, authors do not specify the part of hippocampus that has been studied (DH or VH), but we assume that it is mainly DH.

Severe stressful experience during the last week of pregnancy (immobilization or foot shocks) leads to long-lasting changes of the properties of synaptic plasticity in different brain areas, in particular, in hippocampus of offspring of both genders. Thus, PS favors low-frequency stimulation-induced LTD and inhibits the high-frequency stimulation-induced LTP without affecting basal synaptic transmission in the hippocampus of young (3- or 5-week-old) rats [49–51] as well as in the frontal cortex of adult (3-month-old) animals [52]. Fostering of PS offspring by nonstressed dams to exclude the possible maltreatments of pups by the stressed mother does not abolish the deleterious effects of PS on synaptic plasticity [49, 51]. However, it has been shown that the adoption or postnatal handling can reverse the negative behavioral effects of PS in the adult offspring by altering the activity of the HPA axis and subsequent stress-induced corticosterone release [53, 54].

Yeh and colleagues [51] observed the effects of restraint PS (for 45 minutes three times/day applied at the last week of pregnancy) on synaptic plasticity at young age but the effect disappeared in adult rats (at 8 weeks of age). In contrast, the impairments of hippocampal synaptic plasticity caused by foot shock PS (10 foot shocks/day during the last week of gestation) persist to adulthood (8 weeks of age) in rats as shown by Yang and colleagues [50] but can be cured by an enriched environment treatment at young age. Similar changes of synaptic plasticity after restraint PS during the second week of gestation were seen also in 7-8-week-old male mice [55].

Another approach to PS induction was used by Kinnunen and colleagues [56] as well as by Murmu and colleagues [57]. As shown in [57], the unpredictable stress paradigm consisting of two sessions of three different stressors (restraining in the tube, crowded housing, and forced swim, one on each day during the last week of gestation) is raising blood plasma corticosterone level in pregnant dams and is preventing them from adapting to the stressor. This PS protocol was used in other studies from the same group [58] and was adapted by us [59, 60]. Using this relatively mild stress protocol, Yaka and colleagues [58] were able to show that PS applied during critical period of embryonal development causes deleterious effect on synaptic plasticity of young (4-5-week-old) male offspring expressed as an impaired ability of CA3-CA1 synapses in hippocampus to undergo LTP.

*In vivo* examination of synapses formed by layer 2 of the entorhinal cortex on the granule cells of the DG (the perforant path) of hippocampus showed that short-lasting mild PS (30 min of restraint, from day 15 to day 17 of gestation) leads to facilitation of potentiation of the perforant path in the adult (at 10 weeks of age) offspring [61].

A recent* in vivo* study employed an amplified broad band traffic noise to induce PS and show that either short- (1 h) or long-term (2 or 4 h) exposure to traffic noise affects basal synaptic transmission and impairs posttetanic and long-term potentiation in hippocampus [62]. Rats that were noise stressed for 1 or 2 hours showed deficit in posttetanic phase of potentiation; however, they expressed similar magnitude of LTP at the end of recording session (~2 hours after tetanus). Rats that were prenatally exposed to 4 hours of noise showed constant decline of EPSP slope, which went under baseline values after 2 hours of recording. This could be interpreted as a PS-induced complete loss of ability to express LTP and an appearance of LTD instead. This observation is actually in line with the findings of Gi and colleagues [55] and Yang and colleagues [49] on facilitation of LTD in prenatally stressed animals. Unfortunately, no information on PS impact on synaptic plasticity in VH was provided in these studies.

PS enhances the responsiveness of organisms to acute stress exposure [49, 50, 63–66] via chronic activation of HPA axis that is confirmed by hypertrophy of the adrenal glands [67], which could also underlie the increased vulnerability to develop affective disorders later in life. It has been shown that PS experienced at the third, but not at the second, week of gestation of Sprague-Dawley rats leads to prolonged elevation of the glucocorticoids level in response to acute stress [68]. The alterations in the reactivity of HPA axis in PS rats are correlated with the functional changes of different types of corticosteroid receptors. Thus, PS results in the downregulation of both high-affinity mineralocorticoid receptors (MRs) and low-affinity glucocorticoid receptors (GRs) in rats' offspring [34, 53, 69–71], affecting the binding capacity of MR only [53].

The mechanisms underlying PS impact on hippocampal synaptic plasticity involve tissue plasminogen activator as well as an imbalance in levels of pro- and mature-BDNF (m-BDNF), most likely due to reduced BDNF gene expression and inhibition of conversion of pro-BDNF to m-BDNF [51, 72, 73]. Interestingly, in mouse model for Alzheimer's disease (APPswe/PS1dE9), PS could cause changes in pro- versus m-BDNF levels in hippocampus of 8-month-old female offspring only [74, 75]. Sierksma and colleagues [74, 75] also used chronic restraint stress comparable with [51] but they applied it during the first week of gestation.

Another mechanism that could be involved in processes of regulating synaptic plasticity includes the changes in the functionality of NMDA receptors and their subunits that are important in the induction of both LTP and LTD [76, 77]. The changes in expression of different subunits of the NMDA receptors in different hippocampal fields after PS exposure were shown in [55, 56, 58, 78, 79]. Unlike Yeh and colleagues [51], these studies found that PS not only reduces the levels of NR1 and NR2B subunits but also impairs synaptic localization of the NMDA receptors (low number of complexes associated with PSD95, a NMDA receptor-anchoring molecule). This suggests that PS induces changes in functional activity and distribution of NMDA receptor subunits between DH and VH resulting in different responsivity to stress of the two sectors of hippocampus [38, 39, 45–47, 80].

PS-induced alterations in neurotransmission could underlie the impaired ability to express LTP. In our own studies, we were able to show that PS affects network properties of hippocampal neurons, by reducing GABA-ergic inhibition [59]. PS-induced epigenetic modification of GABA-ergic interneurons not only in hippocampus but also in frontal cortex of young and adult mice mediated by overexpression of DNA methyltransferase associated with a decrease in reelin and GAD67 expression was shown by Matrisciano and colleagues [81]. The alterations in the main excitatory glutamate neurotransmission that are believed to play a role in the pathophysiology of several neuropsychiatric disorders, including schizophrenia, epilepsy, and anxiety, were shown in a study by Marrocco and colleagues [82]. One of the important aspects of that study is the discrimination between the DH and VH. Interestingly, most of their findings were restricted to the VH. Thus, the restraint of dams during the second half of pregnancy (from day 11 until delivery) caused selectivity to VH long-lasting (tested at 3 months of age) reduction of both glutamate release and synaptic vesicle-associated proteins (such as Rab 3A, Munc-18, synaptobrevin, syntaxin-1, synaptophysin, and synapsin) in PS male offspring. The reduction in the activity of mGlu1/mGlu5 receptors in VH of male but not of female offspring was reported earlier by the same group [73]. Interestingly, the deleterious effects of restraint PS on glutamate release in VH as well as some abnormalities in behavior including increased anxiety-like behavior were successfully ameliorated by antidepressants (fluoxetine and agomelatine) [83].

**Figure 2:**
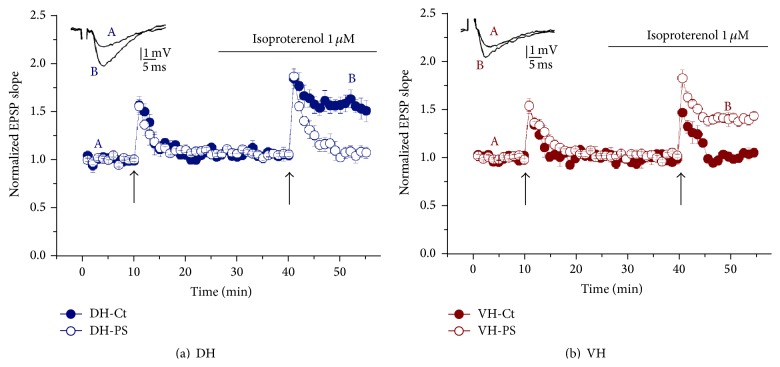
Effect of Isoproterenol, a*β*-adrenergic agonist, on EPSPs recorded in stratum radiatum of DH (a) and VH (b) hippocampal slices from control and prenatally stressed (PS) rats (at 2-3 weeks of gestation). The arrows denote the points at which short tetanic stimulation (35 stimuli at 100 Hz) was delivered, twice to one pathway. Short tetanic stimulation, which normally produces only short-term potentiation, applied in the presence of Isoproterenol, produced a full-blown LTP in DH slices of control group (full circles, (a)) and in VH slices of PS rats (open circles, (b)), but not in the other conditions tested (*adapted from Grigoryan and Segal [60]*).

We also showed that gestational stress in rats selectively modulates noradrenergic (NA) effects in hippocampus of the offspring causing suppression of the ability to convert STP into LTP in the DH and its facilitation in the VH (Figure 2) [60]. An increased plasma noradrenaline level in adult (at 5 months of age) offspring in response to foot shock PS [66] and an impairment in the development of NA neurons in pups from dams exposed to cold stress during the second half of pregnancy were also shown recently [84].

## 4. Ventral Hippocampus and Anxiety-Like Behavior

PS has a lasting effect on the behavior of animals but the data reported by different research groups is conflicting. For review of human studies, see [85, 86]. In the framework of the current review, we will focus on changes in hippocampus-dependent/related behaviors in rodents.* VH* is believed to be involved in anxiety and fear related behavior [19–21, 24, 25, 87]. One of the widely used behavioral tests to assess anxiety in rodents is the elevated plus maze (EPM) test. It is based on a conflict between the rodent's preference for protected area and its motivation to explore novel environments. The avoidance of the open arm by an animal is considered as an anxiety-like behavior [88, 89]. Several studies showed that the exposure to stress during gestation causes changes in emotional status of offspring of both genders. The striking gender-dependent difference in offspring response to PS was exemplified by Zuena and colleagues [73] who showed that males are more prone to developing anxiety-like behavior in the EPM than females that showed reduced anxiety. The increase in anxiety in PS adult offspring (3 months of age) males reported by Zuena and colleagues [73] is consistent with the observations published by the same group [54, 82] as well as with the other studies that utilized traffic noise or varied stressors protocol for prenatal treatments [62, 90, 91]. As to the reduction in anxiety in PS females, it is in disagreement with higher anxiogenic effect of PS in young (5 weeks of age) and young adult (60 days of age) females than in males reported by Salomon and colleagues [90]. In our hands, varying PS during the last week of gestation shifts the emotional balance of young (1 month of age) male offspring into the “less anxious” direction (i.e., PS rats spent more time in the open arm than control rats), and it was correlated with higher motility in the open field and the EPM [59]. Similar effects in young (35 days of age) male offspring as a result of a single but intense PS (120 minutes of maternal immobilization at 16th day of pregnancy) were seen by Cannizzaro and colleagues [92]. Two hours of restraint PS during the second half of pregnancy also leads to more active exploratory behavior in male offspring of the same age [93]. In another study, prenatal restraint stress induced schizophrenic behavior expressed as an increase in locomotion, decreased social interaction, deficit in prepulse inhibition, and contextual fear conditioning was found in adult male rats and male mice [81, 94]. Behavioral profiles indicative of greater emotionality [95] and submissive social rankings [96] were found in the PS offspring of nonhuman primates. In conclusion, the difference in type of maternal stress used as well as the age of the tested animals may lead to the different and sometimes opposite behavioral outcome of PS in the offspring.

## 5. Prenatal Stress and Spatial Learning

The hippocampus, mainly its dorsal part, is believed to be involved in spatial learning and memory processes in both rats and primates [22]. However, both DH and VH support Morris water maze (MWM) spatial learning task, where animals have to learn to navigate to a hidden platform using distal cues [97, 98]. The impaired performance in spatial learning of PS young and adult rats independently of type of stressor used during the last week of pregnancy was shown in a number of studies [50, 55, 62, 94, 99–101]. Using mild stress protocol, Yaka and colleagues [58] and Yang and colleagues [49] were able to show deleterious effect of PS on performance of 4-5-week-old male offspring in MWM. In contrast, in our studies, PS did not impair the behavior of young male rats in MWM learning task and they were actually improving faster than controls during the acquisition phase [59]. The reasons for this discrepancy could be the difference in experimental setup such as a size of the water pool used for the task as well as the training protocol [97]. The facilitation of learning performance in radial maze of mildly prenatally stressed adult (14-15 weeks of age) offspring was shown in [61]. In another study, spatial learning in MWM was not altered by PS in adult (3 months of age) male offspring but leads to an improvement of female offspring performance [73]. In contrast, Wu and colleagues [100] showed impaired performance in MWM of female but not of male offspring. The gender-specific effect of PS on learning in rats as shown by longer escape latencies in MWM in adult (4 months old) and old (12 months old) male but not in female offspring, which was correlated with higher basal corticosterone levels and a lower density of hippocampal corticosteroid receptors in females, was reported in [34, 91]. These observations are supported by the findings of long-term maladaptive behavioral stress responsivity found in mice subjected to PS during the first week of gestation [102]. The anxiogenic behavior and the learning deficit in prenatally stressed offspring are completely abolished by adrenalectomy [91], which confirms the importance of elevated maternal corticosterone in developmental origin of brain vulnerability to PS. Interestingly, repetitive restraint stress during the first week of pregnancy as shown in [75] affects long-term memory acquired in object location task in 7-month-old male mice only, while female offspring shows improved spatial memory performance.

The importance of the timing of PS exposure on learning outcome in adult offspring was shown by Kapoor and colleagues [103]. Thus, the male offspring of guinea pigs that were exposed to PS on gestational days 50, 51, and 52 exhibit impaired spatial learning, while the offspring that was stressed during later phase of* in utero* development (days 60, 61, and 62) appears to exhibit enhanced spatial learning [103].

A somewhat unique study demonstrated effects of PS on cognitive functions in lambs. Prenatally stressed lambs were impaired in a maze performance; they were also characterized by increased fear reactions and pessimistic-like judgment in a cognitive bias test [104].

## 6. Impact of Prenatal Stress on Dorsal and Ventral Hippocampal Morphology

PS influences behavior and memory processes of offspring and it is likely associated with morphological changes in the brain. All studies that have investigated PS-induced changes of brain morphology were focused mainly on DH. To our knowledge, no specific differences of PS effects in DH versus VH have been reported, which leaves an open question of whether PS impacts specifically the morphology of VH.

The age dependence of prenatal restraint stress-induced changes in dendritic morphology of hippocampal pyramidal neurons of areas CA1 and CA3 was shown by Martínez-Téllez and colleagues [93]. They found that CA3 area of the hippocampus is more prone to PS exposure resulting in a decrease in dendritic spine density in prepubertal (at 35 days of age) and adult (at 65 days of age) male offspring, while in the area CA1 the decrease of spine density is characteristic of adult rats only. Interestingly, CA1 pyramidal neurons of hippocampus from prepubertal PS animals were characterized by an increased spine density [93, 105]. In a recent study by Petit and colleagues [106], higher spine density on apical dendrites in the CA1 area of hippocampus of PS lambs immediately after birth was shown [106].

PS during the last week of pregnancy leads to dendritic atrophy expressed as a shortened total length and reduced number of branching points of the apical dendrites of pyramidal neurons of area CA3 of DH also in prepubertal female offspring [78, 105]. In another study that utilized the same stress induction protocol, it was shown that PS does not have an effect either on the total number of neurons or on amount and distribution of both apical and basal dendritic arbors as well as on total spine density of pyramidal neurons of CA1 region of hippocampus in 5-week-old offspring of both genders [51]. Dendritic morphology of CA1 pyramidal cells was not affected in adult (2 months of age) male offspring as well [99].

The decreased synaptic density, length and number of dendritic segments, branching of granule, and CA3 pyramidal hippocampal neurons of young (35-day-old) and adult (2-month-old) male offspring after varied (crowding and daily saline injections during the last week of pregnancy) or restraint PS were demonstrated in other studies as well [99, 107].

PS alters neural and hormonal status also in nonhuman primates as shown in [95]. Thus, 3-year-old male and female offspring of rhesus monkeys that were stressed during early and late periods of their* in utero* development (for 25% of their 24-week gestation, an acoustical startle protocol) were characterized by a 10% reduction in hippocampal volume and inhibition of neurogenesis in the DG associated with increased HPA-axis activity [95]. Prenatal restraint stress as well as varied PS not only induces lifelong reduction of neurogenesis in DG of rat's hippocampus, especially in VH, but also inhibits the facilitation of neurogenesis by learning [67, 108, 109]. However, it was suggested that PS exerts a gender-specific effect on neurogenesis by increasing cell proliferation in the DG of female offspring only [110]. Electron microscopy examination revealed abnormal ultrastructural appearance of hippocampal neurons and myelin sheath in offspring, which was exposed to PS during middle or late stages of embryonal development. In addition, male rats expressed greater impairment than females in these parameters [101]. Moreover, short-lasting mild PS (30 min of restraint, from day 15 to day 17 of gestation) enhances neonatal neurogenesis in hippocampus of 10-week-old male rats, while long-lasting severe PS (240 min of restraint, from day 15 to day 17 of gestation) impairs morphology of hippocampal neurons. Mineralocorticoid and glucocorticoid receptors contribute to PS-induced changes [111].

Morphological analysis of cultured hippocampal cells revealed a reduction in the density of GABA-ergic neurons and the more elaborate dendritic tree of cultured neurons taken from the offspring of PS mothers. However, no difference in dendritic spine density and in the proportion of different spine subtypes was reported [59].

PS causes alterations of neuronal morphology in other young and adult rat brain areas such as nucleus accumbens [93, 112], prefrontal cortex [65, 112], and dorsal anterior cingulate and orbitofrontal cortex [57] as well as corpus callosum of young monkey's brain [113] and prefrontal cortex of brain of newborn lamb [106].

## 7. Conclusions

There are apparent long-term changes in the brain and specifically in the hippocampus following maternal exposure to stress or to stress hormones. These changes are long lasting, as they are caused by epigenetic regulation of gene expression in the brain, as well as by causing stable morphological change in the young, plastic brain. The outcome of these alterations can lead to neurological and psychiatric disorders at a later age. The possible amelioration of the detrimental effects of the adverse stimulation by activation of brain circuits underlying reward and pleasure is now emerging as a promising avenue of repair.
